# Immunomodulatory effects of probiotics and prilled fat supplementation on immune genes expression and lymphocyte proliferation of transition stage Karan Fries cows

**DOI:** 10.14202/vetworld.2018.209-214

**Published:** 2018-02-18

**Authors:** Meeti Punetha, A. K. Roy, H. M. Ajithakumar, Irshad Ahmed Para, Deepanshu Gupta, Mahendra Singh, Jaya Bharati

**Affiliations:** 1 Division of Animal Physiology, National Dairy Research Institute, Karnal - 132 001, Haryana, India; 2 Division of Physiology and Climatology, Indian Veterinary Research Institute, Izzatnagar - 243 122, Bareilly, Uttar Pradesh, India

**Keywords:** beta-hydroxybutyric acid, crossbred cows, dry matter intake, interleukin-1β, lymphocyte proliferation index, prilled fat, *Saccharomyces cerevisiae*, tumor necrosis factor alpha

## Abstract

**Background and Aim:**

Probiotics are the living microorganism which when administered improves the digestion and health of the animal. *Saccharomyces cerevisiae* (SC) improves the humoral and innate immunity of the animal. Prilled fat is a hydrogenated palm oil triglyceride which has been reported to promote the release of cytokines from macrophages. The aim of the study was to evaluate the immunomodulatory effect of probiotic and prilled fat during transition stage in Karan Fries (KF) cows.

**Materials and Methods:**

A total of 12 KF cows at 21 days prepartum were selected and divided into two groups of six animals each. The control group was fed as per the standard feeding practices and the supplemented group cows were supplemented daily with prilled fat at 100 g/cow, SC at 25 g/cow, and sweetener at 1 g/cow in addition to the standard feeding practices from −30 days of prepartum to 21 days of lactation. The sweetener was added to improve the palatability of the feed. The natural sweetener of an African plant leave had 105 times more sweetness than glucose with good aroma. The dry matter intake of the animal was recorded. Plasma samples were collected weekly from all cows for the analysis of blood metabolite beta-hydroxybutyric acid (BHBA). Lymphocytes were isolated from the blood for studying the expression of tumor necrosis factor alpha (TNF-α) and interleukin-1β (IL-1β) and for estimating lymphocyte proliferation index (LPI).

**Results:**

The upregulated IL-1β and TNF-α around calving might be possibly associated to the metabolic changes occurring during the transition period and suggest a higher degree of inflammation around parturition. High concentrations of BHBA caused increased expression and synthesis of the pro-inflammatory factors such as TNF-α and IL-1β in supplemented group in primary calf hepatocytes. The LPI was higher in supplemented group as compared to control which suggests a stimulatory effect of unsaturated fatty acids on mitogen-stimulated T-cell proliferation.

**Conclusion:**

Dietary supplementation of probiotics, prilled fat, and sweetener alleviated negative energy balance by stimulating feed intake and modulating hepatic lipid metabolism; and both of these additives improved the postpartum health (antioxidant status and immune function) of transition dairy cows.

## Introduction

Over the past few decades, production efficiency has increased dramatically due to genetic selection which unfortunately has led to the negative impacts on immunity of animals. Farmers seek natural ways to improve animal health by minimizing the use of antibiotics to achieve higher production. Probiotics in a sense are live, naturally occurring microbes (bacteria and yeast) [1] which when administered in adequate amounts, confers health benefits on the host. Yeast products and their derivatives (i.e., yeast cell wall products) are utilized for a variety of reasons encompassing performance enhancement and overall benefits to animal health and well-being [2]. The transition period between the late pregnancy and early lactation is under a state of natural immunosuppression, which is due to high concentration of plasma cortisol, decreased dry matter intake (DMI), and decreased production of antioxidant defense near parturition, thus leading to the disruption of normal physiology and contributing to periparturient disorders [3]. In general, dairy cows are under a period of negative energy balance (NEB) during the transition period. Excessive NEB results in fat mobilization which in turn increases blood non-esterified fatty acids (NEFA). Large amounts of NEFA are transported to the liver where they undergo β-oxidation in the hepatocytes to generate more ATP to relieve the NEB. This lipid mobilization is accompanied by alterations in inflammatory responses that modify immune function [4].

Previous researches have shown that the unsaturated fatty acids at physiological concentration inhibit proliferation of mitogen-stimulated lymphocyte [5] which may be due to eicosapentaenoate or arachidonate; however, least inhibition was caused by myristate or palmitate.

*Saccharomyces cerevisiae* (SC) is used as a substitute of antibiotic feed additive that increases the DMI. α-D glucan and β-D glucan are the major components of the yeast cell wall which interact directly with immune cell and have antioxidant property [6] which affects immune function [7]. SC supplementation not only enhances digestion and nutritive value but has also resulted in better average daily weight gain, feed conversion rates, and blood constituents [8] which in turn has improved both innate and humoral immunity. Organisms which favor the production of lactic acid producing bacteria in the gut, including SC**, are well known to stimulate various aspects of the immune system, including phagocytic function of macrophages, natural killer cells, monocytes, and neutrophils [9] which are mainly attributed to yeast cell wall components, mainly β glucan and chitin [10]. Dairy calves supplemented with yeast product showed improvement in health and performance [11].

The fatty acid composition of immune and inflammatory cells is sensitive to the changes in fatty acid composition of the diet. The earlier researches have indicated that fatty acid affects the immune response by altering membrane composition and modifying eicosanoids production and cytokine biosynthesis. Animals protect themselves with innate or adaptive mechanism through the production of cytokines and eicosanoids. Dietary fat rich in n-3 and n-6 polyunsaturated fatty acid modulates the inflammatory process. Prilled fat is a hydrogenated palm oil triglyceride consisting of more than 85% palmitic acid which bypasses rumen and does not affect rumen fermentation [12]. These have been reported to promote the release of cytokine from macrophages [13].

Thus, the aim of the present study was to investigate the combined effect of SC and prilled fat on the DMI, beta-hydroxybutyric acid (BHBA), immune gene expression, and lymphocyte proliferation index (LPI) during transition stage in crossbred Karan Fries (KF) cows.

## Materials and Methods

### Ethical approval

The experimental protocol was duly cleared by the National Dairy Research Institute (NDRI), Animal Ethics Committee.

### Experimental site and animals

The NDRI, Karnal, is situated at an altitude of 250 m above mean sea level, latitude and longitude position being 29°42″ N and 79°54″ E, respectively. The maximum ambient temperature in summer goes up to 45°C and minimum temperature in winter comes down to <1°C with a diurnal variation in the order of 15-20°C. For the present study, 12 KF cows at 21 days prepartum were selected from the herd of the NDRI, Karnal.

### Experimental design

The experimental crossbred cows (Holstein Friesian × Thaparkar) were divided into two groups of six animals each on the basis of most probable production ability. Six cows were kept as control and fed as per the standard feeding practices followed at NDRI farm. The six supplemented group cows were supplemented daily with prilled fat at 100 g/cow, SC at 25 g/cow, and sweetener at 1 g/cow in addition to the standard feeding practices from −30 days of prepartum to 21 days of lactation. Plasma samples were collected weekly from all cows for the analysis of blood metabolites (BHBA). Lymphocytes were isolated from the blood for estimating LPI and for studying the expression of TNFα and IL-1β. DMI was recorded on −21, −14, and −7 (prepartum), 0 (parturition), and on day 7, 14, 21 (after parturition).

### LPI

The proliferative response of lymphocytes was analyzed using 3-(4,5-dimethylthiazol-2-yl)-2,5-diphenyltetrazolium bromide (MTT) assay [14]. Blood was collected in heparinized vacutainer tubes and centrifuged at 4°C in refrigerated centrifuge at 3000 rpm for 30 min to separate lymphocytes. The buffy coat at the top was harvested and suspended in 1:1 (v/v) Dulbecco’s phosphate-buffered saline (DPBS). Total contents were carefully layered on lymphocyte separation medium (Histospaque 1077) at the concentration of 3:1 (v/v) in sterile 15 ml polypropylene centrifuge tube and centrifuged at 2000 rpm for 40 min at room temperature. The lymphocyte-rich layer present between plasma and lymphocyte separation medium was collected in another sterile 15 ml polypropylene centrifuge tube having 7 ml DPBS and made volume to 10 ml and centrifuged at 1100 rpm for 10 min. The lymphocyte pellet collected at the bottom of polypropylene centrifuge tube was washed twice with DPBS at 1100 rpm for 10 min. After final wash, culture medium was decanted and cells were resuspended in 3mL culture medium Roswell Park Memorial Institute medium 1640 with 10% fetal calf serum and penicillin G (100 U/ml), streptomycin sulfate (100 µg/ml), and amphotericin B (250 µg/ml). Trypan blue dye exclusion method was used to determine the viability of lymphocytes, and when the lymphocyte viability was above 95%, the lymphocytes were processed further for culture. The cells were adjusted to 1×10^6^ cells per culture well. 200 µl of the diluted cell suspension per well in triplicate was placed in 96 well flat-bottomed tissue culture plate. The mitogen employed in the present study was phytohemagglutinin (PHA-P) at the concentration of 5 µg/ml of the final culture volume (200 µl), a concentration which had been determined previously to provide maximal stimulation of bovine lymphocytes. 20 µl PHA-P was added to the wells. In all the cases, final culture volume was 200 µl. The cells were allowed to proliferate with and without mitogen (PHA-P). The blank wells consisted of 200 µl of culture media only. The culture plates were incubated at 37°C in a humidified CO_2_ incubator (95% air and 5% CO_2_) for 36 h. The proliferative response of lymphocyte was estimated using the colorimetric MTT (tetrazolium). 20 μL of the MTT solution (5 mg/mL dissolved in DPBS and filtered through 0.22 μm Millex-GV filter unit) was added to each well. The plates were again incubated for 4 h at 37°C in humidified CO_2_ incubator. Thereafter, the supernatant was pipetted out completely without disturbing formazan crystal layer, and 150 μL of dimethyl sulfoxide was added to each well. After incubating the plate at room temperature for 15 min, it was shaken on a microplate shaker and the optical density was read using ELISA reader (Microscan MS-5608A) in dual wavelength measuring system, at a wavelength of 570 nm and a reference wavelength of 630 nm. Lymphocyte blastogenic response was expressed as proliferation index (PI) and was calculated as follows:

Proliferation index (PI) = OD of the mitogen-stimulated cells/OD of the non-stimulated cells**.

### Primers

Primers were designed using FAST PCR (version 6.2.73) software details as given in Table-1.

**Table-1:** Target genes, primer sequence (5’--3’), amplicon length, annealing temperature, and EMBL accession number.

Gene	Primer sequence	Size	Tm (°C)	Accession No.
IL1β				
Sense	GAGGAGCATCCTTTCATTCATC	229	56	X54796
Antisense	TTCCTCTCCTTGTACGAAGCTC			
TNFα				
Sense	ACTCAGGTCCTCTTCTCAAGCC	774	56	NM_173966
Antisense	ATGATCCCAAAGTAGACCTGCC			
GAPDH				
Sense	AGCTCATTTCCTGGTACGACAA	184	59	
Antisense	AGGGTCCAGGGACCTTACTC			

IL1β=Interleukin1β, TNFα=Tumor necrosis factor alpha

### Gene expression of TNF-α and IL-1β

Total RNA was isolated from 8 to 10 ml of peripheral blood using the RNeasy Midi Kit (Qiagen), and the concentration and purity of the RNA samples were determined using a NanoDrop. All RNA samples had an A_260/280_ absorbance ratio of between 1.85 and 2.0. RNA undergoes the process of reverse transcription by First Strand cDNA Synthesis Kit (Thermo Science) as the manufacturer recommends. Quantitative real-time polymerase chain reaction (PCR) was performed using SYBR Green, an asymmetrical cyanine dye in PCR Master Mix (Amplicon), primer pairs (Table-1), and an ABI stepOne™ (Applied Biosystems) real-time PCR machine.

### Statistical analysis

The result presented as mean ± standard error of the mean and was analyzed using SPSS (Statistical Package for the Social Sciences) 13.0 software (SPSS Incorporated, Chicago, IL, USA) by two-way analysis of variance followed by Duncan’s multiple range test. p<0.05 is considered statistically significant.

## Results

**Figure-1:**
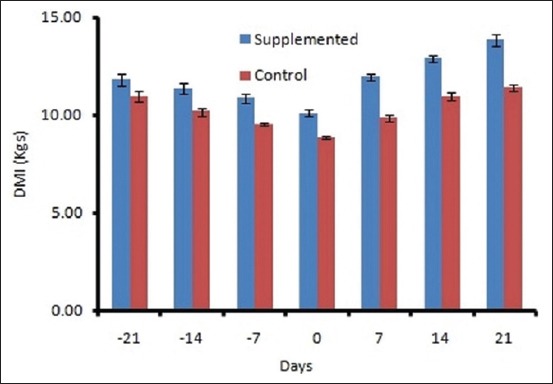
Effect of prilled fat and *Saccharomyces cerevisiae* on Dry Matter Intake in crossbred cows.

**Figure-2:**
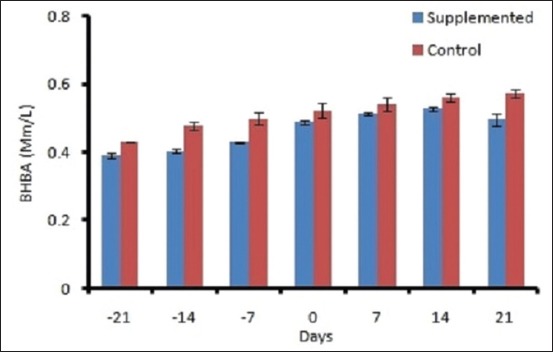
Effect of prilled fat and *Saccharomyces cerevisiae* on Beta Hydroxy butyric acid level in crossbredcows.

**Figure-3:**
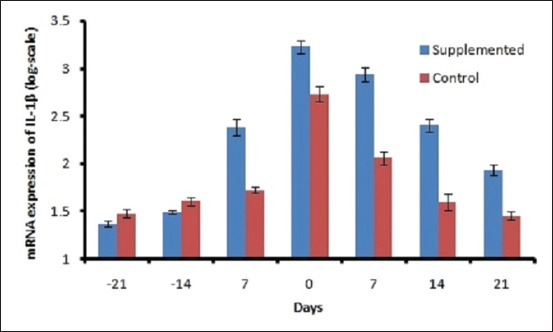
Effect of prilled fat and *Saccharomyces cerevisiae* on mRNA expression of IL-1 beta in crossbred cows.

**Figure-4:**
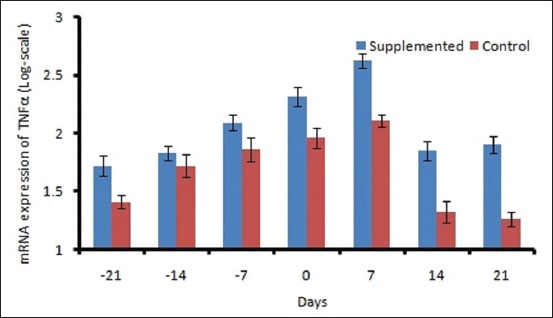
Effect of prilled fat and *Saccharomyces cerevisiae* on mRNA expression of TNFα in cross bred cows.

**Figure-5:**
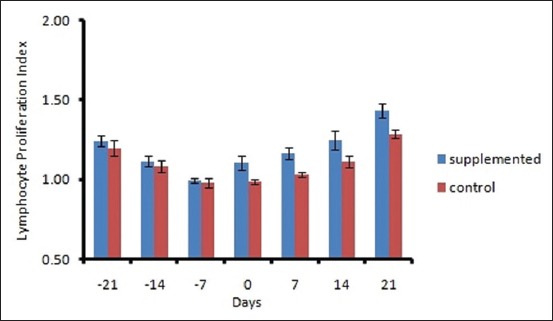
Effect of prilled fat and *Saccharomyces cerevisiae* on Lymphocyte proliferation Index of crossbred cows.

The mean DMI ranged from 10.96±0.29 to 11.4±0.17 kg/d in control and 11.88±0.28 to 13.8±0.29 kg/d in supplemented groups (Figure-1). The DMI varied significantly (p<0.01) between groups and period. A decreasing trend was observed in both the groups during prepartum period from 21 day to calving, and after that, DMI increased linearly. The plasma BHBA concentration varied significantly between the groups. Plasma BHBA concentration ranged between 0.43±0.01 and 0.59± 0.01 mM/L in control and 0.39±0.008 to 0.50±0.01 mM/L in supplemented group during the experimental period (Figure-2). The expression of IL-1β and TNF-α was upregulated during transition period regardless of the diet (Figures-3 and 4). However, supplemented group showed significantly (p<0.01) higher expression than the control group. The LPI was higher in the supplemented group as compared to control with minimum values on 7 day prepartum in both the groups (Figure-5). However, results did not vary significantly between the groups. The average value of LPI varied from 1.20±0.05 to 1.29±-0.03 in control group and 1.24±0.03 to 1.43±0.04 in supplemented group (Figure-5).

## Discussion

The metabolic characteristics of cows during the transition are markedly different from those of other periods. Cows experience serious imbalance in the energy level, amino acids, and other nutrients due to DMI reduction and hormone-induced adaptations [15]. A deficiency in energy intake results in adipose fat mobilization and enhanced activity of lipid metabolic cycles in the liver of dairy cows. Therefore, improving energy status of cow during the transition period is a top priority in dairy nutrition. The DMI of supplemented group was significantly (p<0.01) improved in the following experiment. Yeast was found to improve the feed intake [16,17]. Since prilled fat does not affect DMI [18], the increased DMI in this study was attributed to yeast supplementation as later improve the feed intake [19,20]. Roberts *et al*. [21] conducted an experiment in which animals were fed high energy ration during early lactation and showed lower plasma BHBA concentrations as compared to that of cows on a low energy ration which are in agreement with our study. Yeast supplementation led to the improved energy balance and reduced NEFA level of animals [22]. SC increased the total number of ruminal bacteria and cellulolytic bacteria and the proportion of propionate but decreased lactate concentration and thus increased the DMI of the animal [23]. BHBA concentration declines during the dry period with the increasing DMI [24].

Elevated levels of NEFA and ketone bodies in transition cows are the principal stimulators of immune-suppression, inflammatory responses, and metabolic disorders. IL-1β showed a significant difference between the group and weeks (p<0.01). Production of cytokines such as IL-1β and TNF-α increased in response to pro-inflammatory stimuli [25]. In the current experiment, the upregulated IL-1β and TNF-α around calving could be correlated to the metabolic changes occurring during the transition which suggests a higher degree of inflammation around parturition. Immune mediators including IL-1β and TNF-α play an important role in pathogen clearance [26]. These cytokines stimulate the production of antimicrobial peptides to eradicate the pathogenic bacteria. The higher expression (p<0.01) of IL-1β and TNF-α in supplemented group might be due to increased DMI which resulted in higher glucose availability for cells to respond. BHBA was statistically significant (p<0.01) between the groups. BHBA activates NF-κB-induced hepatocyte injury by regulating the expression of TNF-α and IL-1β in hepatocytes [27]. Cytokines were found to be upregulated significantly in broilers supplemented with SC [28].

Investigations showed that PHA-P stimulated lymphocyte proliferation response on the day of calving and significantly low in all cows. The cell-mediated immune responses were not influenced by the supplement mixture. The supplementation of prilled fat, SC**, and sweetener had no significant effect on the stimulation index of mitogen-induced lymphocyte blastogenesis between control and the supplemented groups. However, the increased value of stimulation index on day 21 of treated group shows that the animals might have recovered from parturition stress. The events involved in lymphocyte activation and proliferation, including signal transduction and receptor expression, are membrane-associated. It is well known that the membrane functions are influenced by the fatty acid composition of the membrane phospholipids, probably because of changes in membrane fluidity. Lymphocytes from different tissues respond differently to dietary lipid manipulation, due to the difference in the ability of different cells to take up and metabolize the fatty acids. The experiment suggests that supplementation stimulated lymphocyte proliferation through the production of IL-1β and TNF-α by stimulated lymphocytes. The yeast extract used in the study contains two bioactive components: Glucan and mannan-oligosaccharides. It was reported that glucan increased total leukocyte number in broiler chicks [29]. However, the LPI in the supplemented group was not significant. Since the effects of fatty acids on lymphocyte proliferation are concentration and time-dependent [5], the variable immunomodulator effects of the yeast extracts may be due to structural differences in glucan [30].

## Conclusion

Dietary supplementation of probiotics, prilled fat, and sweetener alleviated NEB by stimulating feed intake and modulating hepatic lipid metabolism, and both of these additives improved the postpartum health (antioxidant status and immune function) of transition dairy cows. The DMI was improved in the supplemented group when compared with control group. BHBA concentration was lower in the supplemented group which shows improved energy status of animal. The lymphocyte proliferation was higher in supplemented group than the control which has led to higher expression of IL-1β and TNFα. In conclusion, the results showed that the probiotics and prilled fat together improved the immune status of the animals.

## Authors’ Contributions

MP carried out the experiment, analyzed the data and drafted the manuscript. AKR and MS designed the experiment and also supervised the laboratory work. AKH, IP, and DG helped in conducting research and contributed to statistical analysis. MP and JB helped to draft the manuscript. JB, MP, and IP reviewed the manuscript efficiently. All authors read and approved the final manuscript.
